# Has the Gratuité policy reduced inequities in geographic access to antenatal care in Burkina Faso? Evidence from facility-based data from 2014 to 2022

**DOI:** 10.3389/fgwh.2024.1345438

**Published:** 2024-03-21

**Authors:** Marie-Jeanne Offosse, Pierre Yameogo, André Lin Ouedraogo, Zanga Traoré, Aduragbemi Banke-Thomas

**Affiliations:** ^1^ Country Office, ThinkWell Institute, Ouagadougou, Burkina Faso; ^2^ Technical Secretariat for Health Financing Reforms, Ministry of Health, Ouagadougou, Burkina Faso; ^3^ Institute for Disease Modeling, Bill and Melinda Gates Foundation, Seattle, WA, United States; ^4^ Maternal, Adolescent, Reproductive and Child Health Centre, London School of Hygiene and Tropical Medicine, London, United Kingdom

**Keywords:** user fee, health policy, universal health coverage, antenatal care, geographic accessibility, Burkina Faso

## Abstract

**Background:**

Evidence shows that user fee exemption policies improve the use of maternal, newborn, and child health (MNCH) services. However, addressing the cost of care is only one barrier to accessing MNCH services. Poor geographic accessibility relating to distance is another. Our objective in this study was to assess the effect of a user fee exemption policy in Burkina Faso (Gratuité) on antenatal care (ANC) use, considering distance to health facilities.

**Methods:**

We conducted a cross-sectional study with sub-analysis by intervention period to compare utilization of ANC services (outcome of interest) in pregnant women who used the service in the context of the Gratuité user fee exemption policy and those who did not, in Manga district, Burkina Faso. Dependent variables included were socio-demographic characteristics, obstetric history, and distance to the lower-level health facility (known as Centre de Santé et Promotion Sociale) in which care was sort. Univariate, bivariate, and multivariate analyses were performed across the entire population, within those who used ANC before the policy and after its inception.

**Results:**

For women who used services before the Gratuité policy was introduced, those living 5–9 km were almost twice (OR = 1.94; 95% CI: 1.17–3.21) more likely to have their first ANC visit (ANC1) in the first trimester compared to those living <5 km of the nearest health facility. After the policy was introduced, women living 5–9 km and >10 km from the nearest facility were almost twice (OR = 1.86; 95% CI: 1.14–3.05) and over twice (OR = 2.04; 95% CI: 1.20–3.48) more likely respectively to use ANC1 in the first trimester compared to those living within 5 km of the nearest health facility. Also, women living over 10 km from the nearest facility were 1.29 times (OR = 1.29; 95% CI: 1.00–1.66) more likely to have 4+ ANC than those living less than 5 km from the nearest health facility.

**Conclusions:**

Insofar as the financial barrier to ANC has been lifted and the geographical barrier reduced for the populations that live farther away from services through the Gratuité policy, then the Burkinabé government must make efforts to sustain the policy and ensure that benefits of the policy reach the targeted and its gains maximized.

## Background

Despite the significant progress made over the 15 years of the Millennium Development Goals ending 2015 and the ensuing almost nine years of the Sustainable Development Goals till date, the burden of maternal morbidity and mortality remains high in sub-Saharan Africa (SSA) (1, 2). Of the 287,000 women who died due to complications of pregnancy and childbirth in 2020, 70% lived in SSA (1). The maternal mortality ratio (MMR) in Burkina Faso is 264 per 100,000 live births, with about 2,000 maternal deaths reported annually, making it one of the top 15 countries in SSA in terms of annual maternal deaths (1). Access to maternal, newborn, and child health (MNCH) services reduces the risk of adverse pregnancy outcomes such as maternal mortality. Of these MNCH services, antenatal care (ANC) offers an opportunity for skilled health personnel (SHP) to engage with pregnant women, monitor pregnancy, and minimise the risk of adverse pregnancy outcomes (3). As per guidelines published by the World Health Organization (WHO) in early 2000s, four ANC visits were recommended during pregnancy, with the first expected during the first trimester (T1). This guideline was refreshed to eight visits in 2016 with the aim of ensuring a positive pregnancy experience for women (3, 4).

Even at the time of the recommended minimum of four visits, many challenges were reported to affect access and use of ANC services by women in Burkina Faso, including the cost of care, which creates a financial barrier (5). To overcome the financial barrier to accessing and using ANC and other MNCH services, Burkina Faso piloted an initiative to waive the co-payment of services targeting pregnant women and their babies in public health facilities in the year 2000. In 2016, the country scaled up the policy nationally over two months and formally adopted a national policy of free MNCH services, institutionalized within a policy called Gratuité (6). Such user fee exemption policies had long been viewed as critical for the realization of universal health coverage (UHC) in SSA (7). Seven years later, the Gratuité policy is now being implemented in all public and some private health facilities across the country. As per the design, public health facilities provide a defined package of MNCH services free at the point of use to service users. The policy's long-term vision is to significantly reduce avoidable deaths among children aged 0–5 years and women (8, 9).

There is a growing body of evidence showing the impact of the Gratuité policy on improving the use of MNCH services in Burkina Faso (10–12). Similar evidence relating to the positive effect of user fee exemption policies on increasing service utilization in other low- and middle-income countries has also been published (13–15). However, cost of care is only one barrier to accessing MNCH services, poor geographic accessibility relating to distance and travel time is another. Indeed, cost of care and distance to facilities that can provide the needed care are intrinsically linked (16). Evidence shows that distance may either make women not use MNCH services or arrive late with potentially life-threatening problems (17, 18). As such, it is essential to understand if policies such as Gratuité aimed at minimizing the burden related to the cost of care also stimulate service users who live farther away from health facilities to seek care. This is an issue of equity and is more so important in the context of Burkina Faso, where around one in six women live more than 10 km from a health facility. In addition, in a third of the 70 districts in the country, at least two out of three women live more than 10 km from the nearest health facility (19). Our objective in this study was to assess the effect of the Gratuité policy on the use of ANC considering distance to health facilities. The key hypothesis underpinning this objective was that use of ANC amongst a group of women who used the service after the launch of the Gratuité policy would be better than amongst those who used it before the policy was introduced.

## Methods

### Study design

We conducted a cross-sectional study with sub-analysis by intervention period to compare utilization of ANC services amongst pregnant women who used the service in the context of the Gratuité user fee exemption policy and those who did not in a selected district of Burkina Faso. This design was the only feasible option as the intervention was rolled out nationally rapidly (6). As such, there was no opportunity to account for the counterfactual of what would have happened without the intervention.

### Study setting

**Figure 1:**
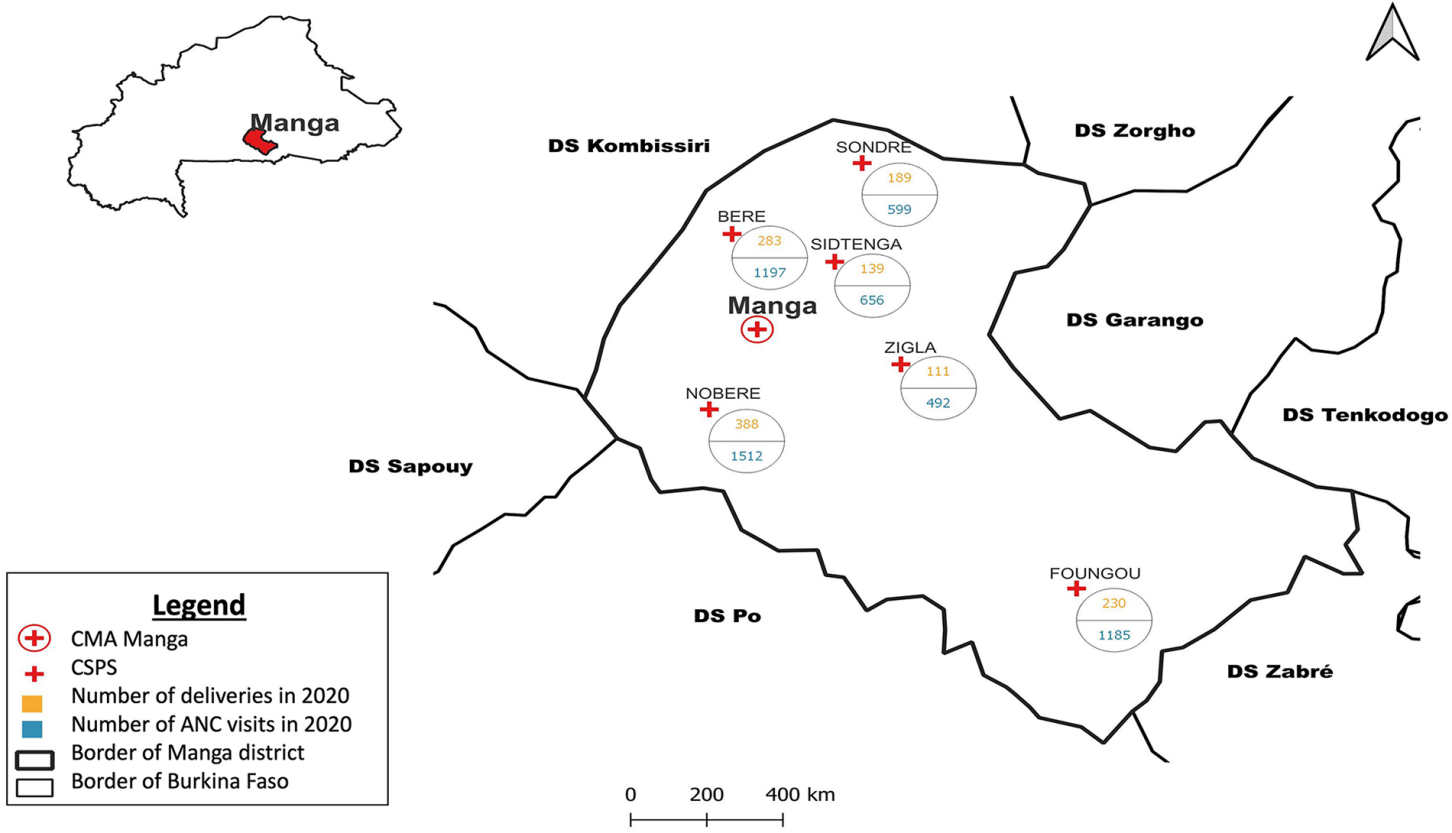
Map of Manga with the selected health facilities geolocated.

Burkina Faso is a landlocked country in West Africa with a population of about 22 million in 2021 and a life expectancy of 60 years. An estimated 41% of the population lives below the national poverty line of US$1.90 daily. The country comprises 13 regions and 63 health districts, each with one district or regional hospital. The study took place in the Centre-South region of Burkina Faso. As per a 2018 survey, rate of completion of at least four ANC visits during the pregnancy (ANC4+), by region ranged from 32.8% to 79.8%, with coverage in the Centre-South at 74.6% (20). Within the Centre-South region, we selected the health district of Manga, which has a total population of 323,628, with 55.1%, 18.3%, and 29.2% living at a distance of 0–4 km, 5–9 km, and over 10 km from a health facility (21). The selection of the district of Manga, which is predominantly rural, where two out of six women live more than 10 km from health centers, and where public transport is more limited (compared to large cities), offered an opportunity to document the effect of the Gratuité policy on women's use of ANC services amongst rural populations. This reasoned choice reflects the reality of Burkina Faso, where 74% of the population lives in rural areas, with around 20% living more than 10 km from health centers (22). Following a preliminary assessment of data completion and quality, the study focused on six of the 35 health facilities [referred to locally as Centre de Santé et Promotion Sociale (CSPS)] in Manga, all public facilities. There are no private facilities in the district. Specifically, we focused on those with data on ANC use and delivery services from 2014 to 2022, except for 2016 (launch of the Gratuité policy) and 2019 (lack of data due to SHP unrest). The selected CSPS were those of Sondre, Sidtenga, Guere, Foungou, Nobere and Zigla (Figure 1).

### Data extraction

We extracted data on monthly indicators of ANC service utilization, relevant and available socio-demographic characteristics, obstetric history, and distance to care for all women listed in the clinic registers for ANC and delivery of the six selected CSPS. Distance to care was based on the recorded straight-line distance estimates from the self-reported village of origin to the health facility of care, routinely reported as a continuous variable. ANC service utilization, our outcome of interest, has been used as a proxy indicator of access in similar studies (10, 23). All data was collected electronically using tablets in the field by research assistants trained for this purpose. A pre-tested survey questionnaire was programmed on Android-based tablets using the Kobo Collect application version v2022.2.3 for the data extraction. The data extracted by the teams of data extractors were synchronized on a remote server after an initial quality control check at the source by a co-data extractor. A data manager, who also served as supervisor to the data extractors, also carried out a second-level quality check to identify any errors that might have escaped the initial field check before uploading the data daily to the server and providing the central coordination team with a progress report. A compilation of errors was sent to the data extractors in the form of field feedback to minimize future errors and improve the efficiency of the data extraction process. Efforts were made to ensure that only single observations were extracted from the registers to avoid duplicate entries.

### Study variables

The dependent variables were the attendance of the first antenatal visit (ANC1) during the T1, as extracted from the ANC register and ANC4+ from the delivery register. The independent variables of interest extracted from both registers were age group (15–19 years, 20–24 years, 25–34 years, and 35 years and above, based on reproductive age risk), marital status (single or married), woman's sector of activity (housewife, student, informal sector, formal sector, and others), distance group travelled to the nearest health facility (0–4 km, 5–9 km, and 10 km or more, in line with the classification used by the government of Burkina Faso), gravidity (the number of times that a woman has been pregnant. Women were classed as primigravida, i.e., the index pregnancy is their first or multigravida, i.e., been pregnant previously), and parity (the number of times that she has given birth to a fetus with a gestational age of 24 weeks or more, regardless of whether the child was born alive or was stillborn. Women could be nulliparous (0), primiparous (1), or multiparous (>1)). These variables were selected because they have been shown to have the potential to explain variation in the outcome indicators of interest in previous studies (16, 24) and they were available in the register.

### Ethical considerations

Ethical approval was obtained from the National Health Research Ethics Committee of Burkina Faso (No2022-05-096). To ensure confidentiality, the research assistants who are SHP themselves were trained in the ethics of data extraction and data transferred to the server was immediately anonymized.

### Data analysis

After data cleaning, which involved correcting outliers, missing values, and duplicates, we recoded the variables of interest as needed for the analysis. Categorical variables were analyzed as frequencies and proportions and presented in tables. A bivariate analysis was performed between the dependent variables (outcomes) and the independent variables (marital status, woman's main activity, and the explanatory variable of interest—distance to care). Associations between independent and dependent variables were tested at a 95% confidence interval (CI), with a significance *p*-value set at ≤0.05. Subsequently, a multivariate analysis was used to explore predictors of using ANC1 at T1 and ANC4+ in the context of the Gratuité policy. The analysis was used to test the association of distance to care as the explanatory variable of interest with ANC1 at T1 and ANC4+ while controlling for other independent variables that were statistically significant. To establish the impact of the policy on the use of services by women living near (<5 km) or far (5–9 km; ≥10 km) from the health facility, we performed logistic regressions based on three samples, i.e., the entire sample accumulating visits before and after the Gratuité policy (Model 1), a sub-sample of visits that occurred before/without the Gratuité policy (Model 2) and a sub-sample of visits with the Gratuité policy (Model 3), including control variables (age, marital status, principal sector of activity of the woman, as well as gravidity and parity of the woman) to avoid bias in the analysis of the exploratory variable of interest. Analysis was done in STATA SE 17.0 (StataCorp, College Station, Texas, United States), ensuring that the objectives of the study were realized.

## Results

A total of 7,281 ANC visits were extracted from the ANC and delivery registries. From both registries, most visits captured were done with the Gratuité policy implemented (55.0% in ANC and 60.6% in delivery). The sample included mostly women aged 25–34 years (39.4% in ANC and 38.6% in delivery), those living less than 5 km from the nearest health facility (41.6% in ANC and 38.3% in delivery), married (94.7% in ANC and 99.6% in delivery), and housewives (61.7% in ANC and 96.9% in delivery). In addition, the sample mostly included women who were multiparous (66.3% in ANC and 64.0% in delivery) and those who had been pregnant previously (78.3% in both registries) (Table 1).

**Table 1:** Socio-demographic and economic characteristics of all women included by register.

Variables	Delivery		Antenatal care	
	*N*	%	*N*	%
Gratuité policy				
Without policy	998	39.4%	913	45.0%
With policy	1,536	60.6%	1,117	55.0%
Age				
15–19	727	16.9%	549	18.5%
20–24 years old	1,125	26.1%	714	24.0%
Aged 25–34	1,660	38.6%	1,171	39.4%
35 and over	793	18.4%	542	18.2%
Distance travelled				
0–4 km	1,652	38.3%	1,237	41.6%
5–9 km	1,460	33.9%	753	25.3%
10 km or more	1,193	27.7%	986	33.1%
Marital status				
Married	4,132	99.6%	2,817	94.7%
Single	17	0.4%	159	5.3%
Main activity				
Housewife	3,853	96.9%	1,551	61.7%
Student	30	0.7%	17	0.7%
Informal sector	74	1.9%	450	17.9%
Formal sector	5	0.1%	12	0.5%
Other	15	0.4%	486	19.3%
Parity				
Nulliparous	761	17.7%	485	16.3%
Primiparous	789	18.3%	518	17.4%
Multiparous	2,755	64.0%	1,973	66.3%
Gravidity				
Primigravida	935	21.7%	646	21.7%
Multigravida	3,370	78.3%	2,330	78.3%
Total	4,305	100%	2,976	100%

From the ANC register, 42.84% (449/1,048) of women completed ANC1 during the first trimester in a CSPS, all periods combined. Without the Gratuité policy [i.e., before its introduction (2014–2015)], this figure was 34.3% (163/449); with the policy, it was 65.7% (286/449). The bivariate analysis showed a significant association (*p* ≤ 0.05) between the explanatory variables, Gratuité and distance travelled to care, with the use of ANC1 in the first trimester. Also, marital status and the woman's main activity were associated with the use of ANC1 in the first trimester. On the other hand, from the delivery register, 59.9% (1,496/2,497) of women had four or more ANC visits in CSPS during all periods combined. Without the Gratuité policy, this number was 35.0% (523/1,496)); with the policy, it increased to 65.0% (973/1,496). The bivariate analysis only indicated a significant association (*p* ≤ 0.05) with Gratuité and not with distance. Also, parity and gestational age were associated with the completion of ANC4+ (Table 2).

**Table 2:** First trimester ANC1 and socio-demographic variables of all women included.

Variables	ANC1 during the first trimester					At least four ANC visits (ANC4+)				
	Yes	%	No	%	*p-*value	Yes	%	No	%	*p-*value
Gratuité policy					0.000***					0.000***
Before	163	34.3%	312	65.7%		523	52.6%	471	47.4%	
During	286	49.9%	287	50.1%		973	64.7%	530	35.3%	
Age					0.615					0.566
15–19 years old	92	43.6%	119	56.4%		287	62.8%	170	37.2%	
20–24 years old	115	45.1%	140	54.9%		388	59.6%	263	40.4%	
25–34 years old	171	40.4%	252	59.6%		561	58.9%	391	41.1%	
35+	71	44.7%	88	55.4%		260	59.5%	177	40.5%	
Marital status					0.018**					0.614
Married	412	41.9%	571	58.1%		1442	59.7%	958	40.3%	
Bachelor	37	56.9%	28	43.1%		8	53.3%	7	46.7%	
Occupation					0.003***					0.192
Housewife	199	41.8%	277	58.2%		1367	59.6%	912	40.4%	
Student/Pupil	6	100.0%	0	0.0%		5	71.4%	1	28.6%	
Informal sector	137	41.9%	190	58.1%		32	60.4%	21	39.6%	
Formal sector	2	100.0%	0	0.0%		1	33.3%	2	66.7%	
Others	83	51.9%	77	48.1%		9	90.0%	1	10.0%	
Distance traveled					0.000***					0.172
0–4 km	109	34.2%	210	65.8%		522	57.9%	379	42.1%	
5–9 km	193	54.5%	161	45.5%		456	59.6%	309	40.4%	
10 km or more	147	39.2%	228	60.8%		518	62.3%	313	37.7%	
Parity					0.853					0.023**
Nulliparous	80	44.7%	99	55.3%		295	65.7%	153	34.3%	
Primiparous	84	42.9%	112	57.1%		282	58.4%	201	41.6%	
Multiparous	285	42.4%	388	57.7%		921	58.7%	647	41.3%	
Gravidity					0.205					0.000***
Primigravida	95	46.8%	108	53.2%		377	67.9%	178	32.1%	
Multigravida	354	41.9%	491	58.1%		1119	57.6%	823	42.4%	

The “Other” category includes young women who have dropped out of school and are not living in a household and housekeepers.

* <0.1 but >0.05.

** <0.05 but >0.01.

*** <0.01.

From the multivariate analysis, presented in Table 3, the global model showed that the odds of seeking ANC1 in the first trimester is more than twice (OR* *=* *2.17; 95% CI: 1.66–2.82) for women who sought care after Gratuité was introduced compared to those before the policy was introduced. From the model that included only women who used services before the Gratuité policy was introduced, women living between 5 and 9 km were almost twice (OR* *=* *1.94; 95% CI: 1.17–3.21) more likely to use ANC1 in the first trimester compared to those living within 5 km of the nearest health facility and women in the informal sector (OR* *=* *0.07; 95% CI: 0.03–0.16) and other sectors (OR* *=* *0.10; 95% CI: 0.03–0.30) were less likely to use ANC1 in the first trimester compared with housewives. In the model that included only women who used ANC after the Gratuité policy was introduced, women living between 5 and 9 km and those who lived more than 10 km from the nearest facility were almost twice (OR* *=* *1.86; 95% CI: 1.14–3.05) and more than twice (OR* *=* *2.04; 95% CI: 1.20–3.48) more likely respectively to use ANC1 in the first trimester compared to those living within 5 km of the nearest health facility. Also, women in the informal sector (OR* *=* *5.33; 95% CI: 3.46–8.20) and other sectors (OR* *=* *3.85; 95% CI: 2.25–5.71) were less likely to use ANC1 in the first trimester compared with housewives (Table 3).

**Table 3:** Results of multivariate analysis of the variable ANC1 during the first trimester.

Variable	Model 1 (Global): Odds ratio ANC1 at T1 (*n* = 1056)			Model 2 (Without Gratuité policy): Odds ratio ANC1 at T1 (*n *= 450)			Model 3 (With Gratuité policy): Odds ratio ANC1 at T1 (*n *= 606)		
	OR	95% CI	*p*-value	OR	95% CI	*p*-value	OR	95% CI	*p*-value
Gratuité policy									
Without	1								
With	2.17	[1.66–2.82]	0.00***						
Distance travelled									
0–4 km	1			1			1		
5–9 km	1.80	[1.30–2.48]	0.00***	1.94	[1.17–3.21]	0.01**	1.86	[1.14–3.05]	0.00***
10 km or more	0.97	[0.70–1.35]	0.86	1.04	[0.54–2.00]	0.91	2.04	[1.20–3.48]	0.00***
Marital status									
Married	1			1					
Single	1.67	[1.01–2.85]	0.07	2.03	[0.74–5.56]	0.17	1.56	[0.75–3.28]	0.24
Main activity									
Housewife	1			1			1		
Informal sector	0.94	[0.71–1.23]	0.63	0.07	[0.03–0.15]	0.00***	5.33	[3.46–8.20]	0.00***
Other	1.05	[0.72–1.54]	0.79	0.10	[0.03–0.30]	0.00***	3.85	[2.25–5.71]	0.00***

* <0.1 but >0.05.

** <0.05 but >0.01.

*** <0.01.

From the multivariate analysis, presented in Table 4, the global model showed that the odds of having four or more ANC visits is over one and a half (OR* *=* *1.66; 95% CI: 1.41–1.96) times more for women who sought care after Gratuité was introduced compared to those before the policy was introduced. From the model that included only women who used services before the Gratuité policy was introduced, primiparous (OR* *=* *2.32; 95% CI: 1.19–4.54) and multiparous (OR* *=* *3.10; 95% CI: 1.65–5.85) women were more likely respectively to undergo four or more ANC compared with nulliparous women. The multigravida women were 0.22 times (OR* *=* *0.22; 95% CI: 0.13–0.39) less likely to have four or more ANC than the primigravid women. In the adjusted model that included only women who used ANC after the Gratuité policy was introduced, women living more than 10 km from the nearest facility were 1.29 times (OR* *=* *1.29; 95% CI: 1.00–1.66) more likely to have four or more ANC than those living less than 5 km from the nearest health facility (Table 4).

**Table 4:** Results of multivariate analysis of the 4ANC+ variable.

Variable	Model 1 (Global): Odds ratio ANC4+ (*n *= 2534)			Model 2 (Without Gratuité policy): Odds ratio ANC4+ (*n *= 998)			Model 3 (With Gratuité policy): Odds ratio ANC4+ (*n *= 1536)		
	OR	95% CI	*p*-value	OR	95% CI	*p*-value	OR	95% CI	*p*-value
Gratuité policy									
Without	1								
With	1.66	[1.41–1.96]	0.00***						
Distance travelled									
0–4 km	1			1			1		
5–9 km	1.09	[0.90–1.33]	0.38	1.06	[0.77–1.46]	0.78	1.11	[0.86–1.44]	0.40
10 km or more	1.20	[0.99–1.46]	0.06	1.10	[0.81–1.48]	0.81	1.29	[1.00–1.66]	0.05*
Parity									
Nulliparous	1			1			1		
Primiparous	1.51	[0.99–2.30]	0.05*	2.34	[1.20–4.56]	0.01**	0.86	[0.49–1.51]	0.60
Multiparous	1.69	[1.12–2.54]	0.01**	3.11	[1.65–5.86]	0.00***	0.82	[0.46–1.46]	0.50
Gravidity									
Primigravida	1			1			1		
Multigravida	0.44	[0.30–0.64]	0.00***	0.23	[0.13–0.39]	0.00***	0.96	[0.56–1.64]	0.88

* <0.1 but >0.05.

** <0.05 but >0.01.

*** <0.01 .

## Discussion

In this study, we set out to compare the influence of the Gratuité policy on the use of ANC considering distance to health facilities. Our results showed that across the health district of Manga, without the policy, those with travel of 5–9 km to care were almost twice as likely to use ANC in the first trimester compared to those who travelled <5 km. Also, women with travel of >10 km were about 30% more likely to use ANC in the first trimester than those who travelled <5 km. With the Gratuité policy, in addition to travel of 5–9 km to care being almost twice more likely to use ANC in the first trimester compared to those who travelled <5 km, women with travel of >10 km were more than twice as likely to use ANC in the first trimester compared to the those who travelled <5 km. In addition, travel of 5–9 km to care were almost twice as likely to ANC4+ compared to those who travelled <5 km. Comparatively, the odds of ANC utilization in line with recommendation were higher for women who lived farther away from facilities in the context of the Gratuité policy than those who lived farther away from health facilities and used ANC without/before the policy. Typically, we would have expected the inverse, with women living farther away from a health facility being less likely to use services as has been reported in studies conducted in similar rural settings (17, 18). In the context of a user fee exemption policy in Ghana, there was reduced probability of using skilled birth delivery as distance to a health facility increased (25). In a separate study, researchers reported lower ANC4+ use in rural compared to urban areas during the implementation of the user fee exemption policy in Ghana (26). However, it appears that the situation in Manga district might be a unique one as even in the global model that included all women in the sample (those who used care with and without Gratuité), those who lived 5–9 km were about twice more likely to seek ANC early in the first trimester than those living within 5 km. This might have more to do with how the health facilities are situated within the district juxtaposed against where most persons live. It is not unusual to have health facilities in areas that include other public services such as schools and markets, which therefore means there are not enough places of abode in the immediate vicinity of the health facilities (27, 28). Irrespective of the relative location of places of abode and health facilities, it is clear from our findings that there is a comparatively higher strength of association for women who travelled farther to care in the group that used ANC1 during the first trimester or completed ANC4+ with Gratuité policy to those who did same without the policy.

Some of the other variables found to have a statistically significant association in the sub-group of women who used ANC in the context of the Gratuité policy also highlight some additional effects of the policy. This includes the significantly higher use of ANC during the first trimester by women in the informal sector and other categories of work, who were over five and almost four times more likely to use ANC in the first trimester compared with housewives. This is a total reversal of the observation in the sub-group that used ANC before the Gratuité policy was implemented when being in the informal sector and other categories of work were at least 90% less likely to use ANC in the first trimester compared with housewives. While we could not find other studies in Burkina Faso that specifically reported this association to the informal sector, it is known that women who work in this sector are from households with the lowest socioeconomic status (SES). In Kaya health district, which is in the North Central region of Burkina Faso, researchers conducted a survey in 2013 which found that women from households with poor SES were significantly less likely to use ANC in the first trimester than those with very high SES (29). In rural Ghana, women employed, including those self-employed and those working in the informal sector, were three times more likely to initiate ANC early compared to the unemployed (30). The difference in our study compared to the Ghana study might be because we had only those in the informal sector. In any case, we postulate that the reversal that we observed in our study might be because pregnant women in the informal sector are now more positively disposed to use ANC early in the first trimester following the introduction of the Gratuité policy since they do not have to pay for the service from their work pay. In addition, without the Gratuité policy, primiparous and multiparous women were over twice and thrice more likely to use ANC4+ than nulliparous women, while multigravida were almost 80% less likely to use ANC4+. A similar pattern was observed in Tanzania based on data from three sequential Demographic and Health Surveys conducted between 1999 and 2010 (31). However, in our study, no statistically significant association was observed in the group with the Gratuité policy. Again, this observed non-significant difference may be because the policy's free access to ANC services equalizes the ability to access care since financial barriers have been removed, and as such, pregnant women can access care irrespective of their risk profile.

There are some strengths of our paper worth mentioning. First, our analysis of women's ANC use is unique, as it shows an equity dimension to the benefits of the Gratuité policy, which hitherto has been challenging to show because of the immediate national scale-up of the policy and the approach used for data entry in health facility registers in Burkina Faso, which does not allow for unique identification of cases. Second, we used the whole population available from the registers for our analysis, which means we could include all women who engaged with the health service `and for whom data was captured. However, there are some limitations to be considered in interpreting our findings. First, the study was conducted in Manga district, an area with relatively low-security challenges. Our findings could be different if the study was conducted in a district with high security challenges. The fear of facing terrorist attacks during long journeys to health centers could attenuate the effects of cost exemption on the use of ANC1 to T1 and ANC4+ by women living more than 10 km from health facilities. As such, our study may not be generalizable to other parts of the country. Second, we conducted a cross-sectional study that helped us “observe” any difference in service utilization patterns in groups that used ANC before and during the implementation of the policy. However, while we can say that the pattern of the association between our variable of interest (distance) was different for the sub-group that accessed ANC with Gratuité compared to the sub-group that did not, we cannot establish a counterfactual. As such, we cannot necessarily conclude that the observed difference is due to policy on service utilization. Indeed, the differences observed could simply be because of behavior changes of women during the different periods, which had nothing to do with Gratuité implementation, new road construction that improved accessibility from far places, or simply due to chance etc. However, this was the only alternative that was possible with the available data and the lack of a counterfactual that would have allowed us to capture “*what would have happened in the absence of the exposure”* (32). In addition, our analysis was limited by available data, as we were only able to consider two dimensions of access (cost and geographical accessibility). According to Penchansky and Thomas, in addition to those two dimensions, “access” also relates to availability of services, organisation of the service to accept users, and acceptability of care (33). Also, data on certain independent variables documented in the literature as influencing ANC use was unavailable in the health facility registers, e.g., the wife's level of education or the husband's economic activity. Third, we could not incorporate the full period of the Gratuité policy implementation in our analysis, as data was missing for the year 2019.

Also, the Coronavirus Disease 2019 pandemic, which has been shown to have had a negative influence on MNCH service utilization, started in 2020 (34–36), which could also have affected our results. However, the fact that we found a positive association in the sub-group that included women who accessed in the middle of the pandemic suggests that the association might have been stronger otherwise. Finally, we have used straight-line distances as per the available data from the registry. While some authors have highlighted the inability to straight line distances to capture the terrain parameters, the additional level of precision it offers in rural areas and for non-urgent services is largely inconsequential (37, 38).

In terms of policy implications, this research highlights that the Gratuité policy is fostering equity by reducing any geographical barrier to ANC that pregnant women face, especially those who live far away from health facilities. This finding builds on existing evidence which established that the Gratuité policy increased service utilization generally and in conflict-affected areas (10–12). Specifically, the finding supports the notion that by facilitating financial accessibility to MNCH services such as ANC, the Gratuité policy also offsets the gap in geographic access to ANC and consequently improves service utilization. It might also explain recent increases seen in rates of ANC4+ (up to 72%) and ANC during the first trimester (up to 53%) as reported in the 2021 Demographic and Health Survey (39). There is a clear case for the government to sustain the Gratuité policy, on top of the cost of MNHC service provision (40), especially as user fees are a significant barrier to MNCH service utilization (41, 42). The evidence generated from this research can be used to support renewed attention to implementing actions that guarantee the sustainability of the policy. Also, it should be highlighted that transport to care is another financial burden that pregnant women face in accessing MNCH services in LMICs (41, 42). Women who live farther away from services will probably pay more to travel to care. As such, policymakers should not lose sight of this challenge, as any investment made to a user fee exemption scheme like Gratuité, which targets the most vulnerable, helps guarantee more value for money (43). For research, the analysis would benefit from including districts with high security challenges to enable the conclusions to be generalized. In addition, the scope of the analysis of equity in access to MNCH services could be extended to include institutional deliveries and postnatal care, which are equally important in improving outcomes for mothers and their babies. Also, a more comprehensive understanding of the impact of the intervention would be possible using an intervention-focused analytical approach, such as the time series analysis. Finally, qualitative studies could provide a better understanding of the link between cost exemption and the use of ANC services by women living far from health centers.

## Conclusion

Our findings show that with the financial barrier to ANC and other MNCH services having been addressed following the introduction of the Gratuité policy, the influence of geographical barriers to care is also minimized for women who live far away from services. The Burkinabé government must make efforts to sustain the Gratuité policy and ensure that its benefits continue to reach the targeted, especially those who are most vulnerable. The policy certainly presents an important opportunity for progress towards UHC.

## Data Availability

The data analyzed in this study is subject to the following licenses/restrictions: The data supporting this study's findings are available from health facilities patients’ registers. Data sharing does not apply to this article as it contains information that could compromise the privacy of research participants. Requests to access these datasets should be directed to Estelle Dabiré Dembélé, dabiree@yahoo.fr.

## References

[1] WHO UNICEF UNFPA World Bank Group, UNDESA/Population Division. Trends in Maternal Mortality 2000 to 2020: Estimates by WHO, UNICEF, UNFPA, World Bank Group and UNDESA/Population Division. Geneva, Switzerland: World Health Organization (2023).

[2] Alkema L Chou D Hogan D Zhang S Moller A-B Gemmill A Global, regional, and national levels and trends in maternal mortality between 1990 and 2015, with scenario-based projections to 2030: a systematic analysis by the UN maternal mortality estimation inter-agency group. Lancet. (2016) 387:462–74. 10.1016/S0140-6736(15)00838-7 26584737 PMC5515236

[3] Villar J Ba’aqeel H Piaggio G Lumbiganon P Miguel Belizán J Farnot U WHO antenatal care randomised trial for the evaluation of a new model of routine antenatal care. Lancet. (2001) 357:1551–64. 10.1016/s0140-6736(00)04722-x 11377642

[4] World Health Organization. WHO Recommendations on Antenatal Care for a Positive Pregnancy Experience (2016) 1–172. Available online at: https://apps.who.int/iris/bitstream/handle/10665/250796/9789241549912-eng.pdf?sequence=1 (accessed January 30, 2024). 28079998

[5] Atchessi N Ridde V Zunzunegui MV. User fees exemptions alone are not enough to increase indigent use of healthcare services. Health Policy Plan. (2016) 31:674–81. 10.1093/HEAPOL/CZV135 26856363 PMC5886034

[6] Ridde V. From institutionalization of user fees to their abolition in West Africa: a story of pilot projects and public policies. BMC Health Serv Res. (2015) 15:S6. 10.1186/1472-6963-15-S3-S6 26559564 PMC4652517

[7] Ridde V Morestin F. A scoping review of the literature on the abolition of user fees in health care services in Africa. Health Policy Plan. (2011) 26:1–11. 10.1093/heapol/czq021 20547653

[8] Ridde V Yaméogo P. How Burkina Faso used evidence in deciding to launch its policy of free healthcare for children under five and women in 2016. Palgrave Commun. (2018) 4:119. 10.1057/s41599-018-0173-x

[9] Boxshall M Kiendrébéogo JA Kafando Y Tapsoba C Straubinger S Metangmo P-M. An Overview of the User Fee Exemption Policy (Gratuité) in Burkina Faso (2020) 1–69. Available online at: https://thinkwell.global/wp-content/uploads/2020/09/Gratuite-in-Burkina-Faso_18-September-2020.pdf (accessed January 24, 2024).

[10] Offosse MJ Avoka C Yameogo P Manli AR Goumbri A Eboreime E Effectiveness of the gratuité user fee exemption policy on utilization and outcomes of maternal, newborn and child health services in conflict-affected districts of Burkina Faso from 2013 to 2018: a pre-post analysis. Confl Health. (2023) 17:33. 10.1186/S13031-023-00530-Z/FIGURES/3 37415179 PMC10324224

[11] Banke-Thomas A Offosse M-J Yameogo P Manli AR Goumbri A Avoka C Stakeholder perceptions and experiences from the implementation of the gratuité user fee exemption policy in Burkina Faso: a qualitative study. Health Research Policy and Systems. (2023) 21:46. 10.1186/S12961-023-01008-3 37280694 PMC10243699

[12] Debe S Ilboudo PG Kabore L Zoungrana N Gansane A Ridde V Effects of the free healthcare policy on health services’ usage by children under 5 years in Burkina Faso: a controlled interrupted time-series analysis. BMJ Open. (2022) 12:e058077. 10.1186/s13561-023-00443-w 36410840 PMC9680150

[13] Lagarde M Palmer N. The impact of user fees on access to health services in low- and middle-income countries. Cochrane Database Syst Rev. (2011) 2011(4):CD009094. 10.1002/14651858.CD009094 21491414 PMC10025428

[14] Oyugi B Kendall S Peckham S. Effects of free maternal policies on quality and cost of care and outcomes: an integrative review. Prim Health Care Res Dev. (2021) 22:e43. 10.1017/S1463423621000529 34521501 PMC8444462

[15] HattLEMakinenMMadhavanSConlonCM. Effects of user fee exemptions on the provision and use of maternal health services: a review of literature. J Health Popul Nutr. (2013) 31:S67–80. PMCID: PMC402170224992804

[16] Wong KLM Brady OJ Campbell OMR Banke-Thomas A Benova L. Too poor or too far? Partitioning the variability of hospital-based childbirth by poverty and travel time in Kenya, Malawi, Nigeria and Tanzania. Int J Equity Health. (2020) 19:15. 10.1186/s12939-020-1123-y 31992319 PMC6988213

[17] Nesbitt RC Lohela TJ Soremekun S Vesel L Manu A Okyere E The influence of distance and quality of care on place of delivery in rural Ghana. Sci Rep. (2016) 6:30291. 10.1038/srep30291 27506292 PMC4978958

[18] Oldenburg CE Sié A Ouattara M Bountogo M Boudo V Kouanda I Distance to primary care facilities and healthcare utilization for preschool children in rural northwestern Burkina Faso: results from a surveillance cohort. BMC Health Serv Res. (2021) 21:212. 10.1186/s12913-021-06226-5 33750364 PMC7941928

[19] Ministère de l’Économie des Finances et du Développement, Institut National de la Statistique et de la Démographie. Annuaire Statistique 2018 (2019) 1–396. Available online at: http://www.insd.bf/contenu/pub_periodiques/annuaires_stat/Annuaires_stat_nationaux_BF/Annuaire_Statistique_National_2018.pdf (accessed January 30, 2024).

[20] INSD. Enquête Harmonisée sur les Conditions de Vie des Ménages (EHCVM) de 2018: Rapport général (2021) 1–171. Available online at: http://cns.bf/IMG/pdf/ehcvm_2018_rapport_general.pdf (accessed January 30, 2024).

[21] Institut National de la Statistique et de la Démographie. Annuaire Statistique (2021) 1–397. Available online at: https://www.sante.gov.bf/fileadmin/annuaire_2021_mshp.pdf (accessed January 30, 2024).

[22] Institut National de la Statistique et de la Démographie. Cinquième Recensement Général de la Population et de l’Habitation du Burkina Faso 2022:1–136. Available online at: https://www.finances.gov.bf/fileadmin/user_upload/storage/Rapport_resultats_definitifs_RGPH_2019.pdf (accessed January 30, 2024).

[23] Nguyen HT Zombré D Ridde V De Allegri M. The impact of reducing and eliminating user fees on facility-based delivery: a controlled interrupted time series in Burkina Faso. Health Policy Plan. (2018) 33:948–56. 10.1093/heapol/czy077 30256941

[24] Mwase T Brenner S Mazalale J Lohmann J Hamadou S Somda SMA Inequities and their determinants in coverage of maternal health services in Burkina Faso. Int J Equity Health. (2018) 17:58. 10.1186/s12939-018-0770-8 29751836 PMC5948792

[25] Johnson FA Frempong-Ainguah F Padmadas SS. Two decades of maternity care fee exemption policies in Ghana: have they benefited the poor? Health Policy Plan. (2016) 31:46–55. 10.1093/HEAPOL/CZV017 25862731

[26] Ganle JK Parker M Fitzpatrick R Otupiri E. Inequities in accessibility to and utilisation of maternal health services in Ghana after user-fee exemption: a descriptive study. Int J Equity Health. (2014) 13:89. 10.1186/S12939-014-0089-Z/TABLES/10 25388288 PMC4318433

[27] Banke-Thomas A Wright K Collins L. Assessing geographical distribution and accessibility of emergency obstetric care in Sub-Saharan Africa: a systematic review. J Glob Health. (2019) 9:010414. 10.7189/jogh.09.010414 30603080 PMC6304172

[28] Macharia PM Joseph NK Nalwadda GK Mwilike B Banke-Thomas A Benova L Spatial variation and inequities in antenatal care coverage in Kenya, Uganda and mainland Tanzania using model-based geostatistics: a socioeconomic and geographical accessibility lens. BMC Pregnancy Childbirth. (2022) 22:908. 10.1186/s12884-022-05238-1 36474193 PMC9724345

[29] Somé A Baguiya A Coulibaly A Bagnoa V Kouanda S. Prevalence and factors associated with late first antenatal care visit in kaya health district, Burkina Faso. Afr J Reprod Health. (2020) 24:19–26. 10.29063/AJRH2020/V24I2.2 34077088

[30] Kotoh AM Boah M. “No visible signs of pregnancy, no sickness, no antenatal care”: initiation of antenatal care in a rural district in northern Ghana. BMC Public Health. (2019) 19:1094. 10.1186/S12889-019-7400-2 31409306 PMC6693094

[31] Gupta S Yamada G Mpembeni R Frumence G Callaghan-Koru JA Stevenson R Factors associated with four or more antenatal care visits and its decline among pregnant women in Tanzania between 1999 and 2010. PLoS One. (2014) 9:e101893. 10.1371/JOURNAL.PONE.0101893 25036291 PMC4103803

[32] Little RJ Rubin DB. Causal effects in clinical and epidemiological studies via potential outcomes: concepts and analytical approaches. Annu Rev Public Health. (2003) 21:121–45. 10.1146/ANNUREV.PUBLHEALTH.21.1.121 10884949

[33] Penchansky R Thomas JW. The concept of access: definition and relationship to consumer satisfaction. Med Care. (1981) 19:127–40. 10.1097/00005650-198102000-00001 7206846

[34] Banke-Thomas A Semaan A Amongin D Babah O Dioubate N Kikula A A mixed-methods study of maternal health care utilisation in six referral hospitals in four Sub-Saharan African countries before and during the COVID-19 pandemic. BMJ Glob Health. (2022) 7:e008064. 10.1136/bmjgh-2021-008064 35173022 PMC8852239

[35] Semaan A Audet C Huysmans E Afolabi B Assarag B Banke-Thomas A Voices from the frontline: findings from a thematic analysis of a rapid online global survey of maternal and newborn health professionals facing the COVID-19 pandemic. BMJ Glob Health. (2020) 5:e002967. 10.1136/bmjgh-2020-002967 32586891 PMC7335688

[36] Semaan A Banke-Thomas A Amongin D Babah O Dioubate N Kikula A ‘We are not going to shut down, because we cannot postpone pregnancy’: a mixed-methods study of the provision of maternal healthcare in six referral maternity wards in four Sub-Saharan African countries during the COVID-19 pandemic. BMJ Glob Health. (2022) 7:e008063. 10.1136/BMJGH-2021-008063 35144921 PMC8844957

[37] Ludrosky JM Newhouse A Hudnall E Sheree A Perle JG. When a straight line is not the most direct method: an evaluation of straight line versus true distance metrics for patients in rural settings. J Behav Health Serv Res. (2023) 50:214–20. 10.1007/S11414-022-09812-5 35945480 PMC9362975

[38] Boscoe FP Henry KA Zdeb MS. A nationwide comparison of driving distance versus straight-line distance to hospitals. Prof Geogr. (2012) 64:188–96. 10.1080/00330124.2011.583586 PMC3835347 24273346

[39] INSD, ICF. Enquête Démographique et de Santé 2021 (2023). 1–815. Available online at: https://dhsprogram.com/pubs/pdf/FR378/FR378.pdf (accessed January 30, 2024).

[40] Banke-Thomas A Abejirinde I-OO Ayomoh FI Banke-Thomas O Eboreime EA Ameh CA. The cost of maternal health services in low-income and middle-income countries from a provider’s perspective: a systematic review. BMJ Glob Health. (2020) 5:e002371. 10.1136/bmjgh-2020-002371 32565428 PMC7309188

[41] Banke-Thomas A Ayomoh FI Abejirinde I-OO Banke-Thomas O Eboreime EA Ameh CA. Cost of utilising maternal health services in low- and middle-income countries: a systematic review. Int J Health Policy Manag. (2021) 10:564–77. 10.34172/ijhpm.2020.104 32610819 PMC9278371

[42] Banke-Thomas A Makwe CC Balogun M Afolabi BB Alex-Nwangwu TA Ameh CA. Utilization cost of maternity services for childbirth among pregnant women with coronavirus disease 2019 in Nigeria’s epicenter. Int J Gynaecol Obstet. (2021) 152:242–8. 10.1002/ijgo.13436 33098673 PMC9087483

[43] Banke-Thomas A Madaj B Kumar S Ameh C van den Broek N. Assessing value-for-money in maternal and newborn health. BMJ Glob Health. (2017) 2:e000310. 10.1136/bmjgh-2017-000310 29081998 PMC5656121

